# From framework to fitness for the 21st century: How Brazil’s 2025 National Curricular Guidelines recast priorities for training physicians

**DOI:** 10.3389/fmed.2025.1730625

**Published:** 2026-01-08

**Authors:** Bruno B. Andrade, Keiko C. M. Sasaki, Rodrigo C. Menezes, Ricardo L. Luzardo Filho, Kátia M. Avena

**Affiliations:** 1 Operações Acadêmicas, Clariens Educação, São Paulo, Brazil; 2 Laboratório de Pesquisa Clínica e Translacional, Instituto Gonçalo Moniz, Fundação Oswaldo Cruz, Salvador, Brazil; 3 Instituto Monster de Ensino, Assistência, Pesquisa e Desenvolvimento Tecnológico em Saúde, Salvador, Brazil

**Keywords:** Brazil, competency-based education, curriculum, digital health, equity, medical education, medical internship, simulation training

## Abstract

After nearly a decade since the promulgation of Brazil’s National Curricular Guidelines (DCNs) for Medicine, the Ministry of Education of Brazil approved a revised regulatory framework for medical training. The new DCNs preserve central structures from 2014 while explicitly aligning training to digital transformation, programmatic assessment, simulation-based safety, and student wellbeing. This study conducts a comparative documentary analysis of the 2014 and 2025 DCNs, analyzes what changed and why it matters, and map these shifts onto prevailing trends in the Global South and North. We propose a practical roadmap for schools in Brazil to adapt quickly and credibly. Our analysis may be relevant to other systems seeking to reconcile social accountability with rapidly evolving clinical, technological, and educational demands.

## 1 Introduction: Why the DCNs were revised

In September 2025, the Ministry of Education of Brazil approved a new version of the National Curricular Guidelines (*Diretrizes Curriculares Nacionais*, DCNs) for Medicine, marking a defining moment for medical education in the country. This revision came nearly a decade after the 2014 guidelines, which had established a competency-based curriculum centered on the Unified Health System (*Sistema Único de Saúde*, SUS) (1) and aimed to produce generalist, critical, and humanistic physicians. Over the ensuing decade, profound technological, social, and epidemiological transformations reshaped both the practice of medicine and the expectations of medical training, creating pressure for regulatory renewal (2–4).

The reform emerged from an extensive process of public consultation and social participation, coordinated by the National Council of Education (3). The process involved universities, professional associations, health managers, students, and civil society representatives (3). The reform sought to respond not only to new professional competencies, such as digital health literacy, patient safety, and collaborative practice, but also to structural challenges in Brazil’s educational landscape. The rapid expansion of medical schools and student intake over the past decade has raised persistent concerns about training quality, regional inequities, and regulatory oversight. In response, the federal government reinforced accountability mechanisms through instruments such as the National Medical Training Assessment Exam (ENAMED, Brazil’s national exam for medical training evaluation), on-site inspections, and supervision of underperforming institutions (3, 5, 6).

The national debate that informed the revision of Brazil’s medical education guidelines was shaped by governmental bodies and by a broad, coordinated academic movement. A central component of this process was the Rever Project (7), a collaborative initiative led by the Brazilian Association of Medical Education (ABEM), in partnership with the Ministry of Health’s Department of Labor and Education Management in Health (SGTES/MS), the Pan American Health Organization (PAHO/WHO), and with support from the Ministry of Education. Drawing on ABEM’s long-standing experience in engaging medical schools, faculty, preceptors, students, and residents across the country, the project organized regional workshops and national forums that helped identify successful educational practices, strengthen indicators, and build consensus around essential elements of medical training. By emphasizing social accountability, equity, and the public mission of medical education, the Rever Project provided critical technical contributions to the national debate and to the development of proposals for updating Brazil’s National Curriculum Guidelines for Medicine.

Globally, medical education reforms have increasingly converged around competency-based frameworks, seeking to link training outcomes to health-system needs while promoting patient-centered, evidence-informed, and socially accountable practice (8–13). Although frameworks from North America and Europe offer useful points of comparison, they should not be treated as normative benchmarks for Brazilian medical education. The epistemological foundations of the 2025 DCNs are rooted in the SUS, a health system built upon universality, equity and comprehensive care that has generated its own conceptual tradition in health professions education (14, 15). In this regard, Brazil aligns more closely with other experiences from the Global South, such as South Africa and India, where competency-based reforms are shaped by structural inequities, heterogeneous health systems and the need to train physicians for high-vulnerability contexts (16, 17). Brazil’s 2025 DCNs participate in this broader movement while retaining a distinctive orientation through the centrality of the SUS and explicit commitments to social accountability (18–21). These characteristics reflect a long-standing national tradition of linking educational policy to public-health and socially responsive medical training (22–24). This systemic linkage differentiates the Brazilian approach from many high-income settings where alignment between medical education and universal-care public-health objectives tends to be less structural.

This Perspective undertakes a comparative documentary analysis of the 2014 and 2025 DCNs, identifying continuities and innovations, discussing their educational implications, and examining how these regulatory choices align, or diverge, from the broader global landscape of medical education. Given that the 2025 DCNs were only recently approved and medical schools are beginning the initial implementation phase, this Perspective offers an early analytical lens to anticipate challenges, support institutional planning, and inform policy decisions during this critical transition period.

## 2 Approach and analytical framework

This Perspective is based on an interpretive documentary analysis of Brazil’s DCNs for undergraduate medical education, focusing on the 2014 and 2025 versions, originally published in Portuguese in the *Diário Oficial da União* (Federal Official Gazette of Brazil) (2, 3). Rather than testing hypotheses, our aim is to interpret regulatory shifts, contextualize them globally, and derive implications for curriculum governance and institutional practice. Comparisons with international frameworks are used as interpretive comparators, rather than as a formal analytic coding frame in the Methods.

The primary sources were the full official texts of the DCNs (2, 3). In addition, we drew on internationally recognized frameworks for medical education and competency-based policy from both the Global South and North (8–13, 16, 17). To contextualize the Brazilian DCNs within the international landscape, we intentionally selected internationally recognized competency-based medical education frameworks from both the Global North and Global South. These sources were selected purposively based on their global relevance, normative influence, and adoption across medical education systems (not via a systematic review process). Our goal was to contextualize Brazilian choices rather than to exhaustively map the literature.

The documents were read iteratively and compared manually, side-by-side, without software, by two authors with expertise in medical education and policy analysis. We employed a framework-informed documentary comparison, combining deductive and inductive reasoning. Six *a priori* domains, that reflect typical loci of regulatory requirements and quality assurance in undergraduate medical education, guided the analysis: (i) structural parameters (duration, total workload, and internship composition); (ii) competency architecture and graduate profile; (iii) assessment; (iv) learning environments and safety; (v) digital health and data governance; and (vi) student support, wellbeing, inclusion, and belonging. Concise definitions for each domain are provided in Supplementary Table 1. No CAQDAS tools were used.

These domains were derived from: (i) the internal structure of the 2014 DCNs (duration, total workload, internship, and competency triad) (2); and (ii) the National Institute of Educational Studies and Research Anísio Teixeira (INEP) on-site external evaluation instrument for undergraduate Medicine, whose dimensions encompass didactic-pedagogical organization; faculty/preceptorship and faculty development, student support, and physical facilities/safety (25). International frameworks (WFME standards (8); GMC Outcomes for graduates (11); AAMC Core EPAs (10); CanMEDS (9); WHO Universal Health Coverage outcomes (12); the NHS Topol Review (13); NMC India (16); HPCSA South Africa (17) informed interpretive comparisons and were not used as a line-by-line analytic scheme). It was operationalized in three steps: (1) two primary authors independently extracted contrasts directly from the DCN-2014 and DCN-2025 texts; (2) each contrast was cross-checked against the INEP/MEC on-site evaluation instrument; and (3) contrasts were verified for convergence/divergence with the international frameworks listed above. Three co-authors conducted secondary checks. Consensus was reached by all five authors. Cross-framework comparisons appear as interpretive triangulation, not as separate analytic outputs.

Documents discrepancies were discussed and resolved through consensus meetings with all five co-authors, with differences limited to interpretive nuance. Team expertise included clinical teaching/assessment and education policy/document analysis. We did not compute inter-rater agreement statistics because the design relied on documentary comparison with heterogeneous clause lengths. First-pass concordance was high, and residual differences were resolved by negotiated consensus.

For each domain, we analyzed convergences and divergences with global standards and frameworks (8–13, 16, 17). We prioritized documents with national or global scope and the most recent revisions available. This process aimed to contextualize Brazilian regulatory choices within global trends across the Global North and South, without the intention of conducting a systematic review.

As this study relied exclusively on publicly available documents, ethics approval was not required.

## 3 Major changes in Brazil’s 2025 DCNs

The full text of the 2014 and 2025 National Curriculum Guidelines for Medicine (2, 3) were analyzed, allowing the identification of convergences and innovations between them. Based on this analysis, the evolution of Brazil’s National Curriculum Guidelines for Medicine (2014–2025) is presented according to thematic axes and educational implications (Table 1; see Supplementary Table 1 for the full comparative matrix). Each of the following sections details the main modifications observed within each axis, emphasizing their impact on curriculum organization and medical training.

**TABLE 1:** Evolution of Brazil’s National Curriculum Guidelines for Medicine (2014 vs. 2025) by thematic axes and educational implications.

Domain	2014 DCNs	2025 DCNs	Why this matters
Duration and minimum workload	≥6 years; ≥7,200 h	Maintains ≥ 6 years; ≥7,200 h	Preserves training time to reach competence.
Internship composition and areas	≥35% total hours; substantial time in Primary Care and Urgency/Emergency in SUS	Maintains ≥ 35%; 30% divided between Family and Community Medicine and Urgency/Emergency; 70% in Internal Medicine, Surgery, OB/GYN, Pediatrics, and Mental Health, including Public Health, Intensive Care, and Trauma/Orthopedics	Reinforces SUS priorities and incorporates critical areas aligned with patient safety and epidemiological demands.
Internship settings	Up to 25% in external institutions; exceptions allowed with HEI authorization	Maintains the 25% limit, with no exceptions; preference for sites linked to SUS and with accredited residency programs (CNRM); explicit quality and safety criteria	Increases national standardization and ensures quality of clinical training environments.
Supervision and preceptorship	Preceptorship in health services under HEI faculty supervision	Full faculty supervision; continuous pedagogical development for preceptors; institutionalization of NAPED for faculty development	Strengthens academic governance and institutional accountability.
Theoretical workload in internship	Up to 20% theoretical content per area	Reduced to 5%–15% per area	Maintains practical predominance while ensuring evidence-based theoretical integration.
Competency framework and graduate profile	Three domains (Care, Management, Education); early service integration; ICT use and foreign language	Maintains the three domains; details digital competencies (AI, telemedicine, ML, big data), data ethics and governance, collaborative leadership, response to health emergencies, sustainability and climate change, well-being, and belonging; includes NAPED	Updates the graduate profile for 21st-century demands; may increase implementation complexity in resource-limited institutions.
Programmatic Assessment	Integrated assessments; national biennial evaluation	Institutionalized programmatic assessment throughout the curriculum and internship; comprehensive pre-internship summative exam; mandatory feedback and action plans	Enhances accountability and readiness checks at critical transitions.
Learning environments and simulation	Conceptual recognition of protected/simulated environments	Mandated skills & simulation labs; identification and remediation	Safer learning; standardized skill acquisition.
Digital health, AI, and data governance (LGPD)	ICTs mentioned; foreign language proficiency emphasized	Explicit AI/telemedicine/ML/big data; data confidentiality; LGPD ethics; foreign language recommended	Aligns with global digital transformation and governance.
Student wellbeing and inclusion	Emphasis on diversity; no explicit time or structural policies	“Green areas”; institutional wellbeing program; center for inclusion and belonging	Protects learners, improves equity, and sustains performance.
Faculty development	NDE required	NAPED (or equivalent) required	Builds faculty capacity for CBME, feedback, and simulation.
Regulatory governance and accountability *(new thematic axis)*	Guidelines allowed greater local flexibility	Increased detail and monitoring (internship, assessment, learning environments); articulation with external regulatory mechanisms (e.g., national exams, site visits, supervision of underperforming programs)	Provides systemic coherence and reduces variability in course quality nationwide.

AI, artificial intelligence; CBME, competency-based medical education; CNRM, National Commission on Medical Residency; DCNs, National Curriculum Guidelines; HEI, Higher Education Institution; ICTs, information and communication technologies; LGPD, Brazilian General Data Protection Law; ML, machine learning; NAPED, institutional centers responsible for faculty development and pedagogical support; NDE, Core Faculty Committee; OB/GYN, Obstetrics and Gynecology; SUS, Brazilian Unified Health System. A more granular, item-by-item comparative matrix is available in Supplementary Table 1.

### 3.1 General structure of the program: duration, workload, and internship

Both DCNs preserve the minimum workload of 7,200 h over at least 6 years (2, 3). The internship remains a central component, corresponding to a minimum of 35% of the total workload, and a duration of 2 years. However, the 2025 update elevates its prominence.

The new DCNs reaffirm the requirement for strong integration of the internship with the SUS, introduce more detailed specifications for clinical rotations, and mandate systematic monitoring of student performance. In addition, they standardize and reduce the theoretical component of each rotation to 5%–15% of total hours. Didactic time should focus on case discussion, evidence-based updating, and supervised consolidation of content essential for safe, supervised clinical practice.

The 2025 DCNs maintain the requirement that at least 30% of internship hours be distributed proportionally between Family and Community Medicine and Emergency/Urgent Care is maintained, rather than equally. The remaining 70% must, in a transversal and integrated manner, encompass Internal Medicine, Surgery, Obstetrics and Gynecology, Pediatrics, and Mental Health, with the explicit inclusion of Public Health, Intensive Care, and Trauma/Orthopedics (3). The 2014 DCNs also orients practical training toward national epidemiological priorities and explicitly incorporates palliative care across the life course.

Another significant improvement concerns the sites where internships may be carried out. The possibility of completing up to 25% of the workload in external institutions was maintained, but exceptions allowing higher percentages with institutional authorization were eliminated. From 2025 onward, eligible external institutions must preferably be linked to SUS, offer medical residency programs accredited by the National Commission for Medical Residency (CNRM) (26), and comply with standards of quality, infrastructure, and safety. Additionally, the new guidelines strengthen the role of the home institution, requiring that its faculty remain actively involved in student supervision even when training occurs in affiliated sites. Together, these changes introduce stricter regulatory standards from 2025 onward, eliminating prior flexibility and establishing a national standard for supervision and quality.

Regarding supervision and preceptorship, both versions acknowledge the importance of service-based teaching in the health system under faculty guidance. The 2025 version, however, advances substantially by instituting continuous pedagogical training programs for preceptors within the health system and by institutionalizing Centers for Pedagogical Support and Faculty Experience (NAPED, *Núcleos de Apoio Pedagógico e Experiência Docente*, structured as institutional centers responsible for faculty development), or equivalent structures, as formal mechanisms for faculty formation and development. The explicit aim is to foster ongoing professional education focused on the pedagogical competencies essential to the “new profile” of physicians.

In practice, from 2025 onward, the internship becomes more standardized, rigorous, and connected to competency-based assessment, whereas in 2014 it was more flexible, descriptive, and open to local adaptation. Points of continuity preserve system integration and large-scale service learning. However, the emphasis on monitoring and standardization in 2025 marks a shift toward greater accountability for medical schools (23), while reducing curricular flexibility.

It is important to underscore the centrality of the assessment system, particularly at the transition to the medical internship. The 2025 Guidelines require at least one comprehensive summative assessment to ensure that students demonstrate the minimum competencies needed for safe, supervised performance in real clinical settings. This measure strengthens institutional accountability and ensures greater uniformity in verifying professional readiness prior to supervised clinical practice.

### 3.2 Competency orientation and graduate profile

The 2014 DCNs formally established a biennial, mandatory national evaluation of medical students, coordinated by INEP (25), designed to be formative and contextual. The results were expected to contribute to the quality assurance of undergraduate programs and could be considered as part of the residency admission process, although its full implementation was never consolidated.

Additionally, the 2014 DCNs established a competency-based curriculum centered on the formation of generalist, humanistic, critical, and reflective physicians, organized into three integrated domains: Health Care, Health Management, and Health Education. This structure promoted early and longitudinal immersion in health services, in addition to the transversal use of information and communication technologies (ICTs) and the requirement to master at least one foreign language (2).

The 2025 DCNs preserve the tripartite structure of medical training, while significantly expanding it by incorporating competencies for the digital era. These include mastery of technological tools such as artificial intelligence (AI), telemedicine, minimally invasive procedures, machine-learning algorithms, large-scale data analytics (Big Data), and artificial neural networks, applied to diagnosis, clinical decision-making, and health management.

In addition, the guidelines mandate compliance with the principles and provisions of Brazil’s General Data Protection Law (LGPD) and related regulations. They require the protection of personal data and the ethical use of information. Security, confidentiality, and integrity must be ensured for personal and sensitive data of patients, professionals, and institutions across all contexts of medical practice.

The new guidelines also incorporate transversal competencies related to sustainability and to action in public-health emergencies and natural or anthropogenic disasters. In these contexts, graduates must demonstrate proficiency in biosafety, health surveillance, risk management, and rapid crisis response, including pandemics, large-scale accidents, and extreme weather events. Moreover, future physicians are expected to understand the social, environmental, and epidemiological impacts of climate change. They should also connect these dimensions to wellbeing, diversity, and a sense of belonging in professional practice.

The 2025 DCNs further reinforce the profession’s commitment to human rights, equity, and diversity, orienting graduates to recognize and value the multiple dimensions of the human condition. It is important to highlight that the guidelines explicitly name the populations historically marginalized within the Brazilian healthcare system. This deliberate act is politically significant because it affirms visibility and strengthens social accountability. The DCNs hallmark the physician’s social responsibility to practice with respect for biological, cultural, ethno-racial, gender, sexual-orientation, and belief diversity, ensuring ethical, inclusive, and humane care, especially for vulnerable or historically neglected populations. These populations include Indigenous peoples; Black communities; Quilombola communities (Afro-Brazilian communities descended from people who established settlements after escaping slavery in Brazil); riverside, rural, and forest populations; people experiencing homelessness; LGBTQIAPN + individuals; people with disabilities; people deprived of liberty; migrants, refugees, and stateless persons, among others. By restoring the full enumeration present in the regulatory text, the guidelines underscore that equity-oriented medical training must be guided by the needs and lived realities of these communities.

The guidelines reaffirm the indissociable integration of teaching, research, and extension, encouraging early scientific engagement and social immersion as part of the graduate profile. Overall, the profile becomes more closely aligned with the demands of the 21st century, incorporating environmental, digital, and psychosocial dimensions and making explicit new expectations: person/family/community-centered care; equity and social justice; collaborative leadership; ethical action in the face of 21st-century sanitary, environmental, and digital challenges; and the critical integration of clinical, scientific, and technological knowledge. Finally, the guidelines underscore an active commitment to the strengthening of the SUS.

The 2025 DCNs reinforce the indissociability of teaching, research, and extension, explicitly promoting early scientific initiation and structured participation in community-based extension projects as formative components of the graduate profile. These initiatives aim to strengthen social accountability and stimulate lifelong learning.

To ensure effective implementation of the goals defined in the 2025 DCNs, medical schools should adopt an integrated set of institutional structures, policies, and practices. These include: a pedagogical support and institutional centers responsible for faculty development (NAPED, or equivalent) as a permanent locus for faculty and preceptor development; an Institutional Center for Inclusion and Belonging responsible for fostering an equitable, welcoming, and diversity-representative educational environment; an Institutional Programmatic Assessment System; an Institutional Student Support and Monitoring Program; a Policy for the Promotion of Student Health and Well-Being; and learning and simulation environments aligned with patient safety and curricular outcomes. While such structures provide a coherent foundation for high-quality medical education, they may pose implementation challenges for institutions with limited infrastructure (5).

### 3.3 Programmatic assessment in medical education

The 2014 DCNs already emphasized integrated assessment processes and provided for a national biennial evaluation coordinated by INEP. However, assessment remained descriptive and locally defined, without a formal programmatic model applied across all curricular components (27). By contrast, the 2025 DCNs transform this paradigm by requiring an institutionalized, continuous, and comprehensive system of assessment, explicitly grounded in the principles of programmatic assessment and encompassing both theoretical and practical components, including the internship. Key indicators and alignment items are summarized in Supplementary Table 1.

As previously detailed, the programmatic assessment (27) integrates multiple sources of evidence on student performance in a longitudinal, competency-oriented model that supports meaningful learning and professional development. The 2025 DCNs consolidate this approach as a national standard for readiness verification. Implementing this methodology requires that medical schools organize an articulated set of tools and occasions, such as workplace-based assessments, objective structured clinical examinations (OSCEs), reflective portfolios, structured feedback, multisource (360°) feedback, and progress tests. Each strategy has limited value in isolation; when integrated within a coherent system, however, they provide a comprehensive view of the learner’s trajectory, increasing the validity and reliability of educational decisions.

To that end, the presence of progression committees (or student monitoring boards) is indispensable. These bodies analyze the aggregate dataset and issue integrated, defensible judgments about the development of competencies defined in the graduate profile. The institutional challenge is to consolidate a system that values competence-based progression, shared pedagogical responsibility, and stewardship of the learner’s developmental pathway.

In addition, the 2025 DCNs require each medical school to implement a comprehensive summative examination immediately before the start of the internship, accompanied by structured feedback and individual action plans in cases of unsatisfactory performance. While the guidelines establish the obligation of a pre-internship readiness assessment, they do not prescribe a national, standardized, or centrally coordinated examination model. The design, scope, and operationalization of this summative exam remain under institutional responsibility. This model points towards more rigorous institutional accountability for medical schools (23) while reducing some aspects of curricular flexibility. Internship assessments likewise undergo substantial change: whereas the 2014 DCNs left the structure of internship evaluation largely to local discretion, the 2025 DCNs strengthen expectations for programmatic assessment and more uniform institutional governance of internship outcomes, without establishing a nationally standardized system.

Student performance must be monitored continuously and linked to the results of the pre-internship summative exam. This ensures that progression is based on objective evidence of competence. In this context, the National Examination for the Evaluation of Medical Training (ENAMED) (6), applied in the 4th and 6th years, has emerged as a federal initiative aimed to support and strengthen programmatic assessment nationwide.

Established in 2025, ENAMED is an annual national exam that evaluates the quality of medical training and holds institutions accountable for students’ academic performance. However, it is not mandated by the 2025 DCNs, which require each school to implement its own programmatic assessment and at least one comprehensive summative exam before the internship (3), without explicitly referring to ENAMED.

In this sense, ENAMED functions as a complementary mechanism that can strengthen institutional evaluation systems. It helps monitor learning progress, identify formative gaps, and verify readiness for supervised practice (5, 6). This reform elevates the internship from a predominantly formative experience to an essential certification milestone. It seeks to consolidate a system-level approach to readiness verification while preserving local curricular identity within national parameters (3, 5, 28–30).

### 3.4 Learning environments and simulation

The 2014 DCNs recognized protected and simulated environments as relevant strategies for teaching and for error analysis; however, they did not require medical schools to maintain dedicated skills or simulation laboratories.

By contrast, the 2025 DCNs make these spaces mandatory, defining them as structured learning environments designed to prepare students for clinical practice with explicit emphasis on patient safety, standardized skill acquisition, and supervised procedural training. Operational elements mapped across both DCNs appear in Supplementary Table 1.

Within these environments, students can err, reflect, and improve without risk to patients, reaffirming that learning safety is inseparable from patient safety. In addition, such spaces must be integrated into assessment and research systems, serving as controlled settings for performance measurement and structured feedback. In this way, infrastructure ceases to be merely physical support and becomes a strategic pedagogical device.

This requirement aligns Brazilian medical training with international standards of safety and quality, recognizing simulation as an essential pedagogical resource for harm prevention and for the progressive development of clinical and communication competencies.

At the same time, the guidelines reaffirm that supervised clinical practice in real healthcare settings remains the irreplaceable core of medical training. Simulation is framed as a complementary strategy that enhances learning safety and supports the gradual acquisition of technical and non-technical skills, but not as a substitute for authentic patient-care experiences within the SUS. The DCNs also clarify that effective simulation does not depend on high-technology or highly aseptic facilities, which may distance students from real-world clinical constraints. Instead, they emphasize low-cost, context-appropriate models grounded in functional fidelity, in which the cognitive, procedural, and decision-making demands of the task outweigh the sophistication of the equipment.

### 3.5 Digital health, AI, and data governance (LGPD)

In 2014, the DCNs merely mentioned ICTs and foreign language proficiency as transversal requirements. In 2025, the text explicitly incorporates AI, telemedicine, machine learning, big data, and the ethics of digital platforms, as well as the protection of confidentiality and personal data in accordance with Brazil’s General Data Protection Law (LGPD) (31). These elements are integrated with evidence-based practice, interprofessional teamwork, and leadership. This update positions Brazilian medical training in direct dialogue with digital transformation and global standards. It aligns with the NHS Topol Review and the WHO outcomes framework, both of which call for digitally literate clinicians capable of working safely in modern information ecosystems (12, 13). The incorporation of digital competencies presupposes the availability of human, technological, and pedagogical resources that remain unevenly distributed across Brazilian schools, requiring phased implementation strategies and institutional centers responsible for faculty development (NAPED). A consolidated comparison of digital-competence requirements is provided in Supplementary Table 1.

### 3.6 Student wellbeing, inclusion, and belonging

The 2014 DCNs emphasized diversity, human rights, and ethical formation, but it did not establish formal policies or structures for student support. By contrast, the 2025 update institutionalizes “green areas” within the standard week, protected time reserved for self-care, research and extension activities, mentoring, and electives (32). It also requires each medical school to implement an institutional program for student support, establish centers for inclusion and belonging, and maintain formal mentoring programs for academic and personal guidance (3). Structural requirements and monitoring suggestions are outlined in Supplementary Table 1.

However, while the 2025 DCNs advance institutional support mechanisms, the broader structural determinants of distress in Brazilian medical training still permeates learning environments. Medical work is still characterized by long hours, productivity pressures and chronic clinical overload, factors consistently associated with high levels of stress, emotional exhaustion and burnout among physicians in Brazil (33, 34). These pressures foster patterns of constant availability, accelerated performance, and early socialization into a culture that normalizes exhaustion. Students are similarly affected, showing marked increases in stress and fatigue during evaluation periods (35, 36) and a persistently high prevalence of common mental disorders linked to intensive workload and institutional pressures even outside crisis contexts (37). Recent analyses of Brazilian medical education further demonstrate how service-centered organizational cultures, workforce shortages, and productivity-driven expectations shape teaching practices and influence both faculty wellbeing and student experience (38).

In parallel, the 2025 DCNs recognizes the importance of inclusive educational environments for historically underrepresented and vulnerable student populations, such as Black, Indigenous, and quilombola students, LGBTQIAPN + individuals, persons with disabilities, and others, through institutional programs for inclusion and belonging. These measures align with national policies for diversity and equity in higher education. The guidelines also extend inclusion to neurodiverse students, promoting accessibility and support mechanisms across the curriculum.

Collectively, the transformations observed in the 2025 DCNs not only reshape the internal dynamics of medical education but also align Brazil’s framework with global competency-based movements that emphasize holistic formation and social accountability. Table 2 presents the alignment of the 2025 DCNs with international frameworks and emerging trends, highlighting convergences with global standards and the distinctive features of the Brazilian context.

**TABLE 2:** Alignment of Brazil’s 2025 National Curriculum Guidelines for Medicine (DCNs 2025) with global frameworks and trends.

Theme	DCNs 2025	Global North exemplars	Global South exemplars	Alignment summary
Programmatic assessment	Required system across the curriculum and internship; comprehensive pre-internship summative exam; mandatory feedback with action plans	WFME standards (8), GMC Outcomes (11), programmatic assessment literature (9, 28–30, 44)	Growing but heterogeneous adoption	Strong alignment; Brazil formalizes readiness checks at transition points
Simulation for patient safety	Mandatory skills and simulation labs with gap identification and remediation	Widely embedded (UK/Canada/US) (8, 9, 11)	Expanding; resource-dependent	Aligned; effectiveness hinges on faculty training and resourcing
Digital health and data governance (AI/telemedicine/ML/big data; LGPD)	Explicit digital competencies; confidentiality/data ethics aligned with LGPD	Topol Review (13); GMC digital expectations (11)	WHO UHC competencies frameworks; national variants (12)	Aligned; governance and privacy explicitly foregrounded
Social accountability and primary care orientation	SUS-centered training; 30% of internship in FCM + Emergency; community-based learning	Present but often less central in curricula	Core in many reforms (India, South Africa) (16, 17)	Brazil remains a pacesetter for primary-care-led accountability
EPAs/observable competence	Pre-internship readiness verification; continuous intern monitoring within programmatic assessment	AAMC Core EPAs (10), GMC transition outcomes (11)	Pilots/adaptations underway	Convergent direction; emphasis on observable competence at transitions.
Student wellbeing, inclusion and belonging	Institutional structures (wellbeing program, inclusion/belonging centers) and “green areas” (protected time)	Increasing emphasis (policies and supports)	Growing recognition	Brazil explicit and structural moves beyond rhetorical commitments.
Faculty development	NAPED (or equivalent) required; continuous pedagogical development, feedback & simulation capacity	CPD frameworks; simulation/assessment educator standards	Variable formalization	Aligned; capacity-building is made a regulatory expectation.
Sustainability and climate change	Explicit competencies on sustainability and climate-health impacts	Emerging (e.g., UK GMC guidance updates) (11, 13)	Increasing in national reforms	Forward-leaning for the region; aligns with global recommendations.
Regulatory governance and accountability	More detailed national monitoring (internship, assessment, learning environments); articulation with external mechanisms (exams/site visits/supervision)	External quality assurance common	Strengthening quality assurance mechanisms	System-level coherence; reduces unwarranted variability across schools.

AAMC, Association of American Medical Colleges; AI, artificial intelligence; CPD, Continuing Professional Development; DCNs, National Curriculum Guidelines; EPAs, Entrustable Professional Activities; FCM, Family and Community Medicine; GMC, General Medical Council; LGPD, Brazilian General Data Protection Law; ML, machine learning; NAPED, Center for Pedagogical Support and Faculty Development; SUS, Brazilian Unified Health System; UHC, Universal Health Coverage; UK, United Kingdom; US, United States; WFME, World Federation for Medical Education; WHO, World Health Organization.

## 4 Implications for medical education and policy

Implementing the 2025 DCNs will require not only structural adjustments but also pedagogical innovation. Active learning methodologies and student-centered approaches are essential to foster autonomy, critical reasoning, and engagement in scientific and extension activities. These strategies aim to ensure that curricular transformation translates into genuine professional competence.

The 2025 revision of Brazil’s DCNs for Medicine represents a significant inflection point in the governance, structure, and ethos of medical education. The innovations presented reveal a movement from normative flexibility toward regulatory consolidation, anchored in the principles of competency-based medical education (CBME), patient safety, and social accountability. These frameworks are mobilized as interpretive comparators to situate Brazil’s regulatory choices globally. They were not used as a line-by-line analytic coding scheme.

Compared with the 2014 framework, the new DCNs articulate a more prescriptive and performance-oriented model, marked by tighter integration with the SUS, standardized assessment practices, and institutional accountability mechanisms. At the same time, the 2025 DCNs expose persistent tensions between national homogenization and local adaptability. The stronger emphasis on monitoring, documentation, and quality assurance may enhance transparency and comparability across medical schools, yet it risks constraining curricular innovation and widening the implementation gap between resource-rich and resource-limited institutions.

To mitigate these disparities, the implementation of the 2025 National Curriculum Guidelines (DCNs) could be supported through coordinated funding models and collaborative resource-sharing mechanisms (8). Regional consortia of medical schools, especially those linked to public universities and teaching networks affiliated with the Brazilian Unified Health System (SUS), could pool infrastructure for simulation, faculty development, and assessment (22, 23). National agencies such as the Ministry of Education (MEC), CAPES, and CNPq might establish targeted funding lines for simulation laboratories and digital platforms, with proportional allocation favoring institutions in resource-limited regions (22, 24). Furthermore, academic consortia and interinstitutional mentoring programs could facilitate knowledge transfer and shared access to pedagogical innovations, thus reducing structural asymmetries.

Balancing compliance with flexibility will be essential for the effective implementation of the 2025 DCNs. Institutions may preserve spaces for pedagogical innovation by integrating elective modules, interprofessional activities, and short innovation cycles within the standard curricular structure. The guidelines’ emphasis on “green areas,” electives, and community-based projects already provides opportunities for experimentation without compromising national standards. Encouraging faculty development in curriculum design and recognizing local pedagogical innovations through national dissemination networks could further sustain creativity while ensuring alignment with regulatory requirements (23, 39, 40).

Among the most significant advances is the explicit acknowledgment of student distress and the requirement for systemic support. These measures move beyond rhetorical commitments by embedding structural time allocations and institutional responsibilities that promote equity, mental health, and identity development. This represents a qualitative leap for Brazilian medical education, aligning it with international calls for learner wellbeing as an integral component of professional competence. However, the transformative potential of these initiatives depends on their authentic implementation; there remains a risk that schools will adopt them as formal compliance measures rather than substantive cultural change. Effectiveness, however, will hinge on measurable uptake and equity of access; schools should track protected-time utilization, counseling access, time-to-support, and differential effects across student subgroups to avoid tokenistic compliance.

Equally transformative is the mandatory establishment of simulation and skills laboratories, which positions Brazil alongside countries where simulation-based education serves as a cornerstone for patient safety and progressive skills acquisition. This minimizes avoidable harm and ensures progressive competence development prior to real-world patient care (3, 8). Yet, despite their pedagogical value, these facilities demand significant financial and infrastructural investment, posing particular challenges for smaller or resource-limited institutions, potentially widening disparities in training quality across the country.

### 4.1 Positioning Brazil’s 2025 DCNs globally

Brazil’s 2025 DCNs can be interpreted as a maturation of CBME, less concerned with enumerating content and more focused on ensuring readiness, safeguarding patients, and caring for learners. The move to a system-wide programmatic assessment, coupled with a comprehensive pre-internship summative exam and mandatory feedback with action plans, brings Brazil closer to the design logic found in WFME standards, GMC Outcomes, and the programmatic assessment literature (8, 9, 11, 28–30).

Although the new requirements present challenges related to faculty development and the standardization of assessment criteria, this regulatory shift nonetheless positions Brazil in alignment with global trends in competency-based medical education, consistent with standards set by WFME (8), GMC (11), AAMC EPAs (10), and CanMEDS (9). Across these frameworks, assessment is conceived as a longitudinal process that integrates formative and summative judgments, emphasizes direct observation of competencies, and uses performance data to guide feedback and remediation. In particular, the WFME (8) standards advocate for coherent, outcome-aligned systems with continuous improvement, while the GMC Outcomes (11), AAMC EPAs (10), and CanMEDS (9) roles converge in prioritizing observable competencies at critical transitions in training.

From a Global North perspective, convergence is evident around observable competence (e.g., AAMC Core EPAs), longitudinal assessment, simulation for patient safety, and digital capability (GMC, WFME, Topol) (8–13, 28–30). Where Brazil diverges is in its explicit social-accountability mandate and SUS orientation, which place health-system stewardship more centrally than in many high-income frameworks (2, 3).

A plausible explanation for these contrasts lies in health-system design and governance. Brazil’s publicly funded SUS (1) embeds legal and regulatory mandates for social accountability, equity, and primary-care orientation, which pull the DCNs toward system stewardship (workforce distribution, primary-care exposure, public-health readiness) as a central educational outcome (2, 3). By contrast, many mixed or insurance-based systems in high-income settings emphasize institutional autonomy and accreditation logics that privilege observable competence, patient-safety simulation, and digital capability while locating social accountability more variably across schools and regions) (8–13, 28–30). Differences in regulatory cadence (periodic national guideline updates versus decentralized program standards) and in quality-assurance instruments further shape how similar competency ideals translate into curricular governance, helping to explain convergence on assessment/simulation/digital skills alongside Brazil’s stronger SUS-oriented mandate (2, 3, 8–13, 28–30).

In the Global South, parallels can be drawn with India’s National Medical Commission, particularly the Attitude, Ethics, and Communication module, which explicitly links competency articulation to professional identity formation. Similarly, South Africa’s Health Professions Council (HPCSA) competencies foreground social justice, teamwork, and community responsiveness as central educational goals. Likewise, WHO’s UHC outcomes framework emphasize cross-cutting, system-ready competencies that prepare graduates to navigate complex health systems (12, 16, 17). Within this broader landscape, Brazil’s formalization of protected student time, mandatory simulation infrastructure, and comprehensive programmatic assessment, including the internship, appears comparatively forward-leaning, positioning the country as an emerging reference point for CBME reform in the Global South.

### 4.2 Educational and workforce implications

Building on the DCNs/2025 (3) regulatory shifts, we translate governance updates into day-to-day implementation in medical schools. We assume that practice-readiness rests on a longitudinal assessment system; that its credibility depends on skills/simulation infrastructure focused on safety; that effective formation today requires integrated digital competence under LGPD-aligned data governance; and that sustainable excellence hinges on institutionally owned wellbeing and inclusion with protected time and structured support. By articulating these four axes (assessment, infrastructure, digital, and learner support), we outline operational pathways for implementation and monitoring that acknowledge resource heterogeneity across institutions and the need for equity-oriented rollout (3).

#### 4.2.1 Assessment

Competency-oriented medical education requires the implementation of integrated assessment systems, rather than the fragmented use of isolated instruments. In the 2025 DCNs, to assess means to understand the educational process as a continuum in which professional development is followed longitudinally, reflectively, and based on multiple sources of performance evidence.

Medical schools should therefore build programmatic assessment architectures that articulate diverse methods, such as workplace-based assessments (WBAs), objective structured clinical examinations (OSCEs), reflective portfolios, and multisource (360°) feedback, so as to produce consistent, fair, and transparent judgments. These systems must be underpinned by clear feedback standards, well-defined rubrics, and progression committees responsible for collegial decisions grounded in longitudinal data and multiple evidence sources (8, 9, 11, 28–30, 39).

The programmatic assessment model proposed in the 2025 DCNs aims to make student performance visible over time, enabling early identification of difficulties, guided remediation, and continuous feedback to learning. By integrating formative, summative, and diagnostic dimensions, it fosters an institutional culture in which assessing is also caring for learning, shifting the focus from sanction to the progressive development of professional competence.

Moreover, the model seeks to align local assessment practices with national accountability and quality directives, especially at critical transition points such as entry into the internship and graduation. Coherence between internal assessments and national certifications [e.g., ENAMED (6)] strengthens the transparency, comparability, and credibility of medical education, reinforcing schools’ commitments to excellence, equity, and patient safety (3, 8, 11).

#### 4.2.2 Infrastructure

The credibility of assessment and medical training is inseparable from the structural conditions of learning. In this sense, the 2025 DCNs reposition educational infrastructure as a strategic element for the quality and equity of training, requiring institutions to make ongoing investments in simulation environments, educational technologies, and faculty development.

Skills and clinical simulation laboratories become essential requirements for accreditation and institutional evaluation, functioning as protected learning environments where students can practice technical procedures, clinical communication, teamwork, and decision-making before entering real, high-risk clinical settings. These spaces should operate with structured briefings and debriefings, safety checklists, scenario libraries aligned with learning outcomes, and systems for recording and analyzing performance that feed directly into the programmatic assessment system.

Within this perspective, simulation is understood as both a pedagogical intervention and a patient-safety intervention, promoting integrated development of cognitive, technical, and socioemotional competencies. By allowing error as part of the formative process, simulation reinforces the principle that learning safety is constitutive of care safety.

However, full implementation of this infrastructure demands substantial investment in equipment, digital technologies, and adequate facilities, as well as specialized faculty training. It is essential to prepare teachers and preceptors to plan, conduct, and evaluate simulated activities, mastering facilitation methods and reflective debriefing. Faculty pedagogical preparation thus becomes a precondition for infrastructure to fulfill its educational function, transforming physical resources into active, safe, and meaningful learning environments.

Accordingly, the contemporary view of medical education endorsed by the 2025 DCNs recognizes infrastructure as a strategic pedagogical device, not merely logistical support, but a core pillar of competency-based training, curricular innovation, and the assurance of quality and safety in medical practice (3, 8, 11).

#### 4.2.3 Digital competence

The assessment-infrastructure nexus must be matched by longitudinal digital literacy. Core competencies in AI, telemedicine, machine learning, and big data should be vertically integrated across preclinical and clinical phases, progressing from conceptual fluency to supervised application in authentic tasks (3, 12, 13).

Equally, LGPD-aligned data governance, covering consent, minimization, security, audit trails, and secondary use, must underpin digital learning ecosystems (e-portfolios, learning analytics, simulation logs) so they remain trustworthy sources for programmatic decisions (3, 8, 11, 13). Embedding these capabilities in clerkships and quality-improvement projects ensures that “digital” acts as a practice multiplier, not an elective add-on (12, 13).

#### 4.2.4 Wellbeing and inclusion

Sustainable performance depends on protected time and institutional responsibility. Structured “green areas” for self-care, mentoring, research/extension, and remediation, monitored for equitable access, are prerequisites for retention and excellence, not optional extras (3, 32). Centers for inclusion and belonging, early-warning systems informed by assessment data, and faculty trained to recognize bias and distress create conditions in which diverse learners can thrive.

These policies must be accompanied by structured programs of academic, psychological, and social support, with emphasis on qualified listening, belonging, and the prevention of mental distress. Institutional responsibility for student care thus ceases to be a matter of goodwill and becomes an ethical and pedagogical obligation, fundamental to the sustainability of medical training and to the retention of humanized professionals committed to equity and human rights.

Crucially, these supports close the loop with assessment culture: welbeing policies are most effective when guided by longitudinal evidence, and healthy learning environments, in turn, enhance the validity of observed performance (3, 5, 20).

## 5 Recommendations for Brazilian medical schools

The following recommendations operationalize the frameworks previously described, focusing on institutional implementation. Effective implementation of the 2025 National Curricular Guidelines (DCNs/2025) requires concrete, measurable, and sustainable actions capable of translating normative principles into consistent educational practice.

The recommendations that follow prioritize operational feasibility and the strengthening of academic governance, aligning programmatic assessment, safety in simulation environments, protected time for wellbeing and learning, inclusion and belonging policies, digital literacy, continuous faculty development, and qualified supervision of the medical internship with national expectations for quality and equity, while, at the same time, respecting the diversity and structural heterogeneity of training institutions (3).

### 5.1 Implement programmatic assessment

Building on the earlier description of programmatic assessment, schools should implement a continuous, multi-evidence system that monitors student development and informs academic decisions (3, 8–11, 39) through structured feedback and progression committees (3, 8–11, 39).

To safeguard decision quality, the system should include a Progression Committee responsible for judging longitudinal evidence sets drawn from workplace-based assessments (WBAs), objective structured clinical examinations (OSCEs), reflective portfolios, and multisource feedback, all against explicit criteria and shared performance standards (3, 8–11, 39). In addition, the 2025 DCNs stipulate a pre-internship summative examination accompanied by formal remediation plans to certify readiness for supervised practice; this capstone aligns institutional judgments with national performance criteria and strengthens the transparency, equity, and credibility of the overall assessment process (3, 8–11, 39).

### 5.2 Consolidate simulation readiness

Schools should ensure the implementation of skills and clinical simulation laboratories that progressively integrate technical–procedural training with scenario-based, interprofessional simulations, in alignment with the competencies specified in the curriculum. These environments must operate with safety checklists, structured briefing and debriefing protocols, case libraries mapped to learning outcomes, and ongoing faculty development plans focused on facilitation, scenario conduction, and reflective analysis of simulated practice.

In the 2025 DCNs, simulation is recognized not merely as a teaching method but as a patient-safety intervention that must generate objective evidence of performance and trustworthy assessment data. Systematic records from debriefing sessions should be incorporated into the programmatic assessment system, thereby strengthening feedback loops in the training process, standardizing learning opportunities, and reinforcing an institutional culture of quality and safety (3, 8).

At the same time, although the 2025 DCNs incorporate sustainability and socio-environmental health, they do not explicitly delineate the competencies required for clinical and organizational action in disasters, extreme weather events, or environmental emergencies, domains increasingly emphasized in international medical-education frameworks (41, 42). Given the country’s socio-environmental vulnerabilities and the rising frequency of climate-related crises documented globally (43), simulation laboratories represent an optimal pathway for developing disaster-medicine competencies. Scenario-based simulations involving mass-casualty incidents, floods, heat-related emergencies, or service disruptions can provide structured, safe environments for students to practice crisis decision-making, teamwork, triage, and communication, skills essential for contemporary medical practice in a changing climate.

### 5.3 Operationalize protected time (“green areas”)

Institutionalizing protected time within the curriculum, dedicated to self-care, research and extension activities, academic mentoring, and guided remediation, requires transparent eligibility criteria and systematic monitoring of utilization and equity across student groups. These intervals should be formally embedded as structural components of the pedagogical project, rather than optional periods, to promote mental health, academic engagement, and the sustainability of learning.

To ensure effectiveness and distributive justice, the school should operate tracking dashboards and simple indicators capable of detecting underuse or unequal access and, from there, activate institutional support responses (tutoring, counseling, workload adjustments, research opportunities). By linking student wellbeing policies to concrete educational outcomes, “green areas” consolidate the school’s commitment to the integral and humanized formation of future physicians (3, 32).

### 5.4 Center for Inclusion and Belonging

Establishing and implementing an Institutional Center for Inclusion and Belonging within a medical school is a strategic, structuring action to consolidate an academic culture that is more equitable, welcoming, and representative of human diversity. More than a physical space, the center should function as a pedagogical and relational device capable of mobilizing people, fostering critical reflection, and inducing institutional practices that translate, into everyday academic life, the principles of equity, social justice, and respect for difference.

The center’s mission is to coordinate and articulate institutional policies on inclusion and diversity with emphasis on ethno-racial, cultural, social, gender, and sexual-orientation dimensions, while developing permanent programs for awareness, training, and response to discrimination. At the same time, it should ensure psychosocial welcome, qualified listening, and support for students in situations of vulnerability, acting as a bridge between individual care and institutional transformation.

To make this mission operational, the center must adopt clear governance, maintain dashboards that monitor access, progression, and organizational climate, and provide rapid-response supports (academic, psychosocial, and financial), triggered by early indicators derived from assessment data. These mechanisms should be accompanied by the publication of annual reports to secure transparency, institutional learning, and continuous improvement. By institutionalizing these functions, the Center for Inclusion and Belonging render the medical school’s social accountability tangible and strengthens the retention and success of diverse students (3, 8, 19, 20).

### 5.5 Make digital literacy core

The 2025 DCNs consolidate digital literacy as one of the structuring pillars of medical training, integrating content on artificial intelligence (AI), telemedicine, machine learning, and big-data analytics into clinical cases, supervised clerkships, and health-innovation projects.

Beyond technical proficiency, the guidelines emphasize ethics and data governance in compliance with Brazil’s General Data Protection Law (LGPD), ensuring responsible, secure, and transparent use of information across all educational and care processes. Accordingly, teaching should explicitly address informed consent, data minimization and security, auditability, and responsible reuse, so that e-portfolios, simulation records, and learning-analytics systems function as trustworthy and ethical sources for educational and clinical decision-making (3, 8, 11–13).

This shift requires the Curricular/Program Committee (NDE) to adopt a new mindset toward curricular review, integration of digital technologies into the learning process, and the cultivation of a pedagogical culture capable of preparing physicians to act with competence, critical judgment, and responsibility in the era of digital transformation in health.

### 5.6 Faculty development on a scale

To professionalize teaching, feedback, bias mitigation, and competence in simulation and assessment, schools should implement NAPED (or an equivalent) as a permanent faculty-development structure. This program must include SUS preceptors, strengthening the education–service interface and ensuring competency-based, ethically grounded, and socially accountable training with clear feedback standards across practice settings.

Faculty development should be modular, continuous, and anchored in real practice artifacts, such as rubrics, debriefing notes, and feedback excerpts, to drive consistent and measurable change (3, 8). In this perspective, NAPED or its institutional equivalent stands as a pillar of academic governance, indispensable for sustaining the implementation of the 2025 DCNs and for consolidating a faculty culture oriented toward quality, equity, and innovation in medical education.

### 5.7 Internship governance

Governance of clinical rotations requires standardizing rotation portfolios to ensure compliance with the ≥35% internship requirement and with the mandated exposure to Primary Care and Emergency within the SUS. Local assessments should be aligned with the pre-internship capstone and the programmatic assessment framework, so that judgments at transition points are coherent across training sites.

To sustain comparability, programs should use shared rubrics, set targets for workplace-based assessment coverage, and hold periodic calibration meetings across teams and practice settings, thereby maintaining reliability of results across rotations (2, 3).

## 6 Limitations

We analyzed official regulatory documents and leading international frameworks. Empirical evaluation of implementation fidelity in schools, associated costs, or educational effects will require follow-up studies at institutional and regional levels. Although grounded in current educational policy and international trends, forward-looking statements reflect informed expectations rather than empirically demonstrated outcomes. As with any policy-oriented analysis, the implications discussed here depend on effective implementation, contextual variation, and ongoing monitoring.

Our approach was a framework-informed documentary comparison with manual extraction and consensus verification, rather than a formal qualitative coding study with inter-rater statistics. This may limit replicability. We mitigate this by providing a full clause-level matrix (Supplementary Table 1). International frameworks are mobilized as interpretive comparators, not as a formal coding scheme.

## 7 Conclusion

Brazil’s 2025 DCNs preserve the competency-based foundation anchored in the SUS while modernizing the means of assuring readiness, through programmatic assessment, simulation for patient safety, digital health capability, and structured support for student wellbeing and inclusion. Rather than adding topics, they recalibrate governance over how competence is developed, evidenced, and sustained. Implemented with fidelity, the DCNs have the potential to support Brazil, and peer systems, in preparing physicians for the clinical, technological, and social realities of the next decade.

Conditions for success include: (i) end-to-end programmatic assessment with clear feedback standards and progression committees; (ii) staged simulation readiness with debriefing protocols; (iii) LGPD-compliant data governance for digital learning; (iv) measurable access to wellbeing and inclusion supports; and (v) faculty development at scale via NAPED.

A concise 3-year monitoring set should track: readiness-exam pass rates; coverage and quality of workplace-based assessments; simulation and debriefing throughput; utilization of protected time; and equity in progression and retention. With equity-first rollout and targeted technical support for lower-resourced schools, Brazil can position itself as a Global South reference for competency-based reform aligned with public-system needs.

## Data Availability

Publicly available datasets were analyzed in this study. This data can be downloaded from the Brazilian Ministry of Education: https://www.gov.br/mec/pt-br/cne/normas-classificadas-por-assu nto/diretrizes-curriculares-cursos-de-graduacao.
