# Supplementary material for: From framework to fitness for the 21st century: How Brazil’s 2025 National Curricular Guidelines recast priorities for training physicians
Source: Front Med (Lausanne). 2026 Jan 8;12:1730625. doi: 10.3389/fmed.2025.1730625 (PMC12823480; doi:10.3389/fmed.2025.1730625)
Supplement: Supplementary file 1 [file Data_Sheet_1.PDF]

Supplementary table 1. A priori domains' definitions

Domain	Operational definition	Illustrative content areas
**1. Program structure**	Regulatory parameters defining the organization, workload, and duration of the undergraduate medical course, including internship composition, distribution of practice sites, and supervision requirements.	Total workload (≥ 7,200 h); internship (≥ 35%); Family and Community Medicine/Emergency Care distribution; external site limits; faculty oversight.
**2. Competency in architecture and graduate profile**	Articulation of the physician's competency framework and graduate attributes integrating cognitive, technical, ethical, and social dimensions.	Generalist, critical, and humanistic formation; digital-era competencies (AI, telemedicine, big data); sustainability; social accountability; respect for diversity.
**3. Assessment**	Systems and processes for evaluating learning and verifying readiness for supervised practice, emphasizing programmatic and longitudinal assessment.	Workplace-based assessments (WBAs); OSCEs; portfolios; progress tests; progression committees; pre-internship capstone exam; feedback and remediation plans.
**4. Learning environments and safety**	Standards governing physical, pedagogical, and psychosocial safety in teaching and clinical settings, including simulation for patient and learner protection.	Skills and simulation laboratories; supervision ratios; briefing/debriefing protocols; safety checklists; faculty training (NAPED).
**5. Digital health and data governance**	Expectations for technological competence and ethical data use in alignment with Brazil's General Data Protection Law (LGPD).	AI and machine-learning applications; telemedicine; data security and confidentiality; e-portfolios; learning analytics; audit trails and consent management.
**6. Student support, well-being, inclusion and belonging**	Institutional responsibilities for learner health, mentorship, diversity, and equity in medical education.	"Green windows" (protected time); student support programs; mentoring; centers for inclusion and belonging; early-warning systems; mental-health services.

Table legend: Abbreviations: AI: Artificial Intelligence; LGPD: Brazilian General Data Protection Law; NAPED: institutional centers responsible for faculty development and pedagogical support; OSCEs: objective structured clinical examinations; WBAs: Workplace-based assessments.
